# Genetic Parameter Estimation and Genomic Prediction of Duroc Boars’ Sperm Morphology Abnormalities

**DOI:** 10.3390/ani9100710

**Published:** 2019-09-23

**Authors:** Yunxiang Zhao, Ning Gao, Jian Cheng, Saeed El-Ashram, Lin Zhu, Conglin Zhang, Zhili Li

**Affiliations:** 1 College of Life Science and Engineering, Foshan University, Foshan 528231, China; 2 Department of pig breeding, Guangxi Yangxiang Agriculture and Husbandry Co., LTD, Guigang 537100, China; 3 State Key Laboratory of Biocontrol, School of Life Sciences, Sun Yat-sen University, Guangzhou 510006, China; 4 Animal Science Department, Iowa State University, Ames, IA 50011, USA

**Keywords:** Duroc boars, genetic parameters, genomic prediction, single-step GBLUP, sperm morphology abnormalities

## Abstract

**Simple Summary:**

We estimated genetic parameters and conducted genomic prediction for five types of sperm morphology abnormalities in a large Duroc boar population. Results reflect that the studied traits showed moderate to low heritability and moderate to high repeatability. Both genomic best linear unbiased prediction (GBLUP) and single-step GBLUP (ssGBLUP) showed comparative predictive abilities, and ssGBLUP outperformed GBLUP under many circumstances. To our knowledge, this is the first time that the genetic parameters and genomic predictive ability of sperm morphology abnormalities were reported in such a large Duroc boar population.

**Abstract:**

Artificial insemination (AI) has been used globally as a routine technology in the swine production industry. However, genetic parameters and genomic prediction accuracy of semen traits have seldom been reported. In this study, we estimated genetic parameters and conducted genomic prediction for five types of sperm morphology abnormalities in a large Duroc boar population. The estimated heritability of the studied traits ranged from 0.029 to 0.295. In the random cross-validation scenario, the predictive ability ranged from 0.212 to 0.417 for genomic best linear unbiased prediction (GBLUP) and from 0.249 to 0.565 for single-step GBLUP (ssGBLUP). In the forward prediction scenario, the predictive ability ranged from 0.069 to 0.389 for GBLUP and from 0.085 to 0.483 for ssGBLUP. In conclusion, the studied sperm morphology abnormalities showed moderate to low heritability. Both GBLUP and ssGBLUP showed comparative predictive abilities of breeding values, and ssGBLUP outperformed GBLUP under many circumstances in respect to predictive ability. To our knowledge, this is the first time that the genetic parameters and genomic predictive ability of these traits were reported in such a large Duroc boar population.

## 1. Introduction

Genomic prediction (GP) [1], also termed genomic selection in the fields of animal and plant breeding, has advantages in precisely predicting genetic values, phenotypic values, and disease risks by integrating dense genetic marker panels into prediction models. The basic idea of GP is to trace the effects of all quantitative trait loci (QTL) using dense genomic markers that are in linkage disequilibrium (LD) with the neighbor candidate QTL. Total genetic values can be obtained by summing the effects of all markers [1] or solving mixed model equations (MMEs) with a marker-derived relationship matrix as a covariance matrix [2]. Currently, many methods for either marker effect estimation [1,3,4] or direct genetic value prediction [2,5,6,7] have been established. Among the previously proposed GP approaches, single-step genomic best linear unbiased prediction (ssGBLUP) [5,6] is preferable for genetic evaluation in populations with large numbers of phenotyped individuals but few genotyped individuals, especially in the breeding programs of domesticated animals. The estimated breeding values (EBVs) are calculated by solving MMEs with **H** (a blend of both pedigree- and genetic-marker-derived relationship matrices) as a relatedness matrix. The ssGBLUP approach has been used in the genetic evaluation of pigs [8,9], dairy cattle [10,11], dairy goats [12], and beef cattle [13]. Previous studies have shown that ssGBLUP is perceived to be better or at least comparable in performance to conventional BLUP and the standard GBLUP in respect to predictive ability.

Artificial insemination (AI) has been used globally as a routine technology in the swine production industry. The advantages of elite boars can be disseminated in larger populations rapidly through the implementation of AI. Production of high-quality semen (in terms of boar genetic merits, sperm motility, number of sperm cells per ejaculate, etc.) is essential to guarantee good AI results. Duroc boars have been used as a terminal sire breed in most commercial pig production systems. However, due to small single-boar populations and difficulties in obtaining large-scale measurements of these traits, the genetic parameters and genomic prediction accuracy of economically important semen traits have seldom been reported. Previously, it has been reported that the heritability of boar semen traits is moderate to low [14,15], suggesting the possibility of conducting genetic improvement programs on these traits. Among the usual semen parameters, the proportion of sperm morphology abnormalities (ABN) is a key factor that affects semen quality and fertility. However, the routine assessment of semen morphology abnormalities as a whole may be inadequate for sperm quality evaluation [16]. Using the UltiMateTM CASA system (Hamilton Thorne Inc., Beverly, MA, USA), different types of morphology abnormalities (for detailed definitions, refer to the Materials and Methods section) can be distinguished. Genetic analysis by treating the subtypes as different traits may provide insight into how these abnormalities contribute to sperm quality and fertility.

In this study, we estimated genetic parameters and conducted genomic prediction for five types of sperm morphology abnormalities in a large Duroc boar population. Random cross-validation and forward prediction scenarios were designed to assess the predictive ability or EBV reliability of GBLUP and ssGBLUP. Furthermore, the predictive performance of ssGBLUP was compared to GBLUP.

## 2. Materials and Methods

### 2.1. Ethics Statements

This study was carried out in accordance with the principles of the Basel Declaration and recommendations of the Institutional Animal Care and Use Committee of Foshan University. The protocol was approved by the Institutional Animal Care and Use Committee of Foshan University.

### 2.2. Phenotypes, Genotypes, and Pedigree

The Duroc boar population used in this study was kept in the AI station of Guangxi Yangxiang Co., Ltd., Guigang, China, which is one of the largest boar AI stations in the world. The dataset consisted of 5284 pigs, which can be traced back to 12 generations in the pedigree. Fresh semen of 2693 boars collected from 2015 to 2018 was evaluated using the UltiMateTM CASA system (Hamilton Thorne Inc., Beverly, MA, USA). After ejaculation, 10 µL of fresh semen was diluted 10–30 times (based on the sensory concentration) with a special diluent and 1 µL of the diluted semen was used for evaluation. Among the phenotyped boars, 29,526 observations of morphology abnormality traits for 1304 boars were available and used in this study (Table 1).

The definitions of the five types of sperm morphology abnormalities are described in the CASA system as below:

- PD: Proximal droplet—a droplet of cytoplasm attached to the base of the head where the midpiece emerges.
- DD: Distal droplet—a cytoplasmic droplet attached further down the midpiece/tail from the base of the head. If the droplet is located at more than 4 µm from the base of the head, the droplet is defined as distal. It may indicate an immature sperm and is considered a defect.
- BT: Bent tail—the bending rate of the tail exceeds 20°/µm.
- CT: Coiled tail—the tail bends 180° or more over its length.
- DMR: Distal midpiece reflex—the tail is wrapped around a distal cytoplasmic droplet, typically at the end of the midpiece (close to 4 µm from the end base of the sperm head), and the tail returns to the sperm head, usually becoming visible at the top of the head.

DNA was extracted from a total of 1733 pigs (fresh semen of boars and ear tissues of sows) using genome extraction kits (Wuhan NanoMagBio Technology Co., Ltd., Wuhan, China) and genotyped using the GGP 50k SNP array (GeneSeek, Lincoln, NE, USA), which contains 50,703 SNPs in total. Genotypic data were filtered using Plink software [17] for an individual call rate of ≥90%, an SNP call rate of ≥90%, a minor allele frequency (MAF) of ≥0.01, and a Hardy–Weinberg equilibrium *p*-value of ≥10^−5^. After quality control, 1623 individuals with 36,254 SNPs remained for further analysis. Among the genotyped boars, 1231 had phenotypic data. 

Missing genotypes were simply treated as heterozygotes since the SNPs of unknown physical position were not removed from the dataset, which made LD-based imputation difficult. Considering the low proportion of missing genotypes (0.14%) and the number of markers of unknown physical position (7144), the strategy used in this study may be preferable compared with removing position-unknown SNPs and imputing the missing genotypes.

### 2.3. Statistical Model

In this study, the single-trait repeatability model used was as follows:

**y** = **Xb** + **Za** + **Wp** + *Age* + *Intv* + **e**

where **y** is a vector of phenotypic records; **X**, **Z**, and **W** are incidence matrices associated with **b**, **a**, and **p**, respectively; **b** is a fixed-effect vector (combining the year and season of ejaculation); **a**~N (0, **K**σ_a_^2^) is a vector of the individual additive genetic values; **K** is the relatedness matrix (**H**, **G**, or **A** matrices); σ_a_^2^ is the additive genetic variance; **p**~N(0, **I**σ_p_^2^) is a vector of random permanent environmental effects for each individual; **I** is the identity matrix; σ_p_^2^ is the variance of permanent environmental effects; covariates *Age* and *Intv* are the age of the boar in months at the time of ejaculation and collection interval (days), respectively; **e**~N(0, **I**σ_e_^2^) is a vector of residuals; and σ_e_^2^ is the residual variance. The relationship matrices used in different methods are defined as follows:

**BLUP:** The conventional BLUP [18] model was fitted to obtain the breeding values, which were used for calculating the de-regressed proofs (DRPs) for assessing predictive ability. The pedigree-derived relationship matrix **A** was used as covariance.

**GBLUP:** In the standard GBLUP [2], the relatedness matrix was constructed as ***K**** = **G** = ZZ’/∑^m^_i = 1_2p_i_(1 − p_i_)*, where **Z** is a MAF-adjusted genotype matrix (0–2p, 1–2p, and 2–2p represents AA, Aa, and aa, respectively), *p_i_* is the MAF of the *i*th SNP, and m is the total number of SNPs.

**ssGBLUP:** The inverse of the relatedness matrix **H**, using both pedigree and genetic markers [5,6], can be obtained as follows:

$$K^{-1}=H^{-1}=A^{-1}\;+\;\left[\begin{array}{cc} 0 & 0\\ 0 & G_{\omega }^{-1}-A_{22}^{-1} \end{array}\right]$$

where **A** is the pedigree-based additive genetic relationship matrix, **A**_22_ is a submatrix of **A** for the genotyped individuals, and **G**_w_ = 0.9**G** + 0.1**A**_22_, the weights were the same as our recent paper [19].

### 2.4. Estimation of Genetic Parameters

The variance components were estimated from the full dataset via the same model as Equation (1) using average information restricted maximum likelihood (AI-REML) [20]. The variance component estimation was conducted using the ASReml-R package (VSN International, https://www.vsni.co.uk/). We additionally estimated the genetic correlations among the traits using a multitrait model, where the model is similar to Model (1) except that the response variable was replaced with an *n* × *t* matrix (*n* and *t* are the number of observations and traits, respectively).

### 2.5. Assessment of Genomic Predictive Ability

The breeding value estimations described in this section were performed using the BLUPF90 software family [21].

*DRPs*: In order to assess predictive ability, DRPs with the removal of the parental average (PA) for all individuals in the pedigree (with the exception of parents-unknown individuals) were calculated as described by Garrick et al. [22]. A BLUP model based on Equation (1) with a pedigree-derived numerator relationship matrix as a covariance matrix was solved to obtain the conventional breeding values, which were then converted into DRPs [8,22].

*Cross-validation*: We designed a 5-fold cross-validation to assess the predictive ability of GBLUP and ssGBLUP. In order to ensure comparability between GBLUP and ssGBLUP, only genotyped individuals were included in the test sets. The genotyped individuals were randomly divided into five groups, and each group was treated as a test set once. For GBLUP, the remaining four groups of genotyped individuals were used as training sets. For ssGBLUP, all individuals in the pedigree except those in the test set were used as a training set. The predictive ability was calculated as the Pearson’s correlation between the DRP and the predicted genetic values in the test set. The cross-validation (random grouping among the genotyped individuals and prediction) was repeated 20 times.

*Forward prediction*: In real pig breeding programs, historical records are used to predict genetic values of newly born individuals. Therefore, a forward prediction was designed to assess the predictive ability of different models to mimic the real pig breeding situation. In this scenario, boars from the first to ninth generations were treated as a training set and used to fit the prediction model. Boars from the 10th and 11th generations were treated as a test set and used to validate the model. Table 2 shows the data structure in the forward prediction scenario. Predictive ability was calculated as the Pearson’s correlation between the DRP and the predicted genetic values in the individuals from the 10th and 11th generations. In order to directly compare the EBVs estimated by GBLUP and ssGBLUP, scatter plots of EBVs for individuals from the 10th and 11th generations were additionally drawn.

## 3. Results

The estimated variance components, heritability, and repeatability are shown in Table 3. The estimated heritability (repeatability) values of the traits coiled tail (CT), bent tail (BT), proximal droplet (PD), distal droplet (DD), and distal midpiece reflex (DMR) were 0.029 (0.113), 0.138 (0.298), 0.244 (0.550), 0.295 (0.468), and 0.267 (0.599), respectively. The studied traits showed moderate to low heritability and moderate to high repeatability. Positive correlations between the studied traits, ranging from 0.052 (between PD and DMR) to 0.955 (between BT and CT), were observed (Table 4).

In the random cross-validation scenario, ssGBLUP outperformed GBLUP for all traits in respect to predictive ability (Table 5). The correlations between EBVs from GBLUP (ssGBLUP) and de-regressed proofs were 0.212 (0.249), 0.373 (0.439), 0.330 (0.565), 0.386 (0.507), and 0.417 (0.561) for the traits CT, BT, PD, DD, and DMR, respectively. On average, the predictive ability was improved from 0.344 (ranging from 0.212 to 0.417) to 0.464 (ranging from 0.249 to 0.565) when comparing GBLUP to ssGBLUP. The predictive ability of ssGBLUP was improved by 17.45%, 17.69%, 71.21%, 31.35%, and 34.53% (with an average of 35.10%) for the coiled tail, bent tail, proximal droplet, distal droplet, and distal midpiece reflex, respectively, compared with GBLUP.

In the forward prediction scenario, ssGBLUP outperformed GBLUP in four out of five traits, with the exception of the bent tail, for which the predictive ability of ssGBLUP was similar to that of GBLUP (Table 5). The correlations between EBVs from GBLUP (ssGBLUP) and de-regressed proofs were 0.069 (0.085), 0.311 (0.311), 0.250 (0.311), 0.389 (0.442), and 0.344 (0.483) for the traits CT, BT, PD, DD, and DMR, respectively. On average, the predictive ability was improved from 0.273 (ranging from 0.069 to 0.389) to 0.326 (ranging from 0.085 to 0.483) when comparing GBLUP to ssGBLUP. For the traits coiled tail, proximal droplet, distal droplet, and distal midpiece reflex, the predictive abilities of GBLUP were improved by 23.19%, 24.40%, 13.62%, and 40.41%, respectively, compared with ssGBLUP.

**Figure 1:**
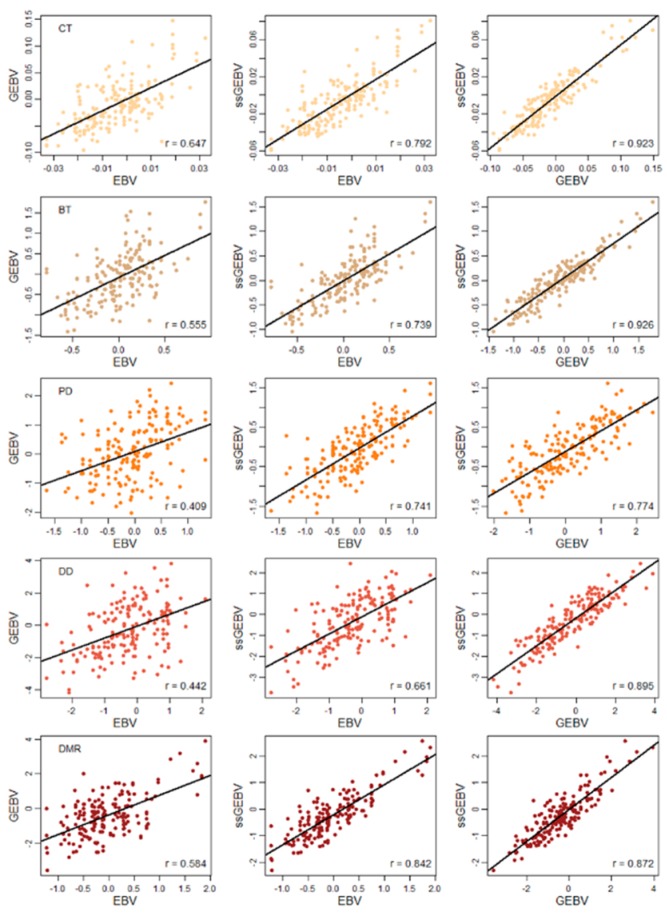
Correlations of estimated breeding values of the test set in the forward prediction scenario. CT: Coiled tail; BT: Bent tail; PD: Proximal droplet; DD: Distal droplet; DMR: Distal midpiece reflex. Each row represents a trait (with the same color).

Figure 1 shows the correlations of estimated breeding values of the test set in the forward prediction. Overall, the correlations increased when comparing EBV versus GEBV to EBV versus ssGEBV and increased when comparing EBV versus ssGEBV to GEBV versus ssGEBV as well. The correlations of EBV versus GEBV, EBV versus ssGEBV, and GEBV versus ssGEBV ranged from 0.409 to 0.647, 0.661 to 0.842, and 0.774 to 0.942, respectively (Figure 1).

## 4. Discussion

In the pork industry, production of high-quality semen depends on different factors including appropriate nutrition, a comfortable environment, good air quality, sufficient movement, and so forth [23]. However, the selection of boars with better genetic backgrounds and semen production management is important to ensure long-term genetic and economic benefits [23,24]. Using low-dose insemination technology [25], the genetic merits of selected elite boars can be rapidly disseminated to large populations. In addition, studies have shown that boar semen traits are heritable, with moderate to low heritability, suggesting that these traits can be genetically improved [14,15]. Currently, dense markers covering the whole genome can be obtained at an acceptable price. Development of novel genomic selection approaches and software has provided a number of opportunities and tools that can be used to implement genomic selection and accelerate genetic improvement of boar semen traits.

We estimated the genetic parameters of five types of sperm morphology abnormalities in a large Duroc boar population. The results showed that the traits were all heritable, though moderate to low heritability was observed (Table 3). The heritability estimates in this study were in the same scale as that for other semen traits in large White and Landrace boars [14,15] and fit well with the usual knowledge regarding the heritability of pig reproductivity traits [26]. These results reflect the complex underlying polygenic background of the studied traits and indicate the possibility of genetic improvement of these traits by selection based on BLUP models. 

The accuracy of the genetic evaluation of sperm morphology abnormalities was further investigated by integrating pedigree, genetic markers, and phenotypic records simultaneously. The ssGBLUP was implemented for the genetic evaluation of the studied sperm morphology abnormalities, with comparison to GBLUP. DRP was calculated and used to evaluate the predictive ability, but it was not used as a response variable in the prediction model. The raw phenotypic records were used as the response variables and the full model was used as the prediction model, which was based on the consideration that using DRP as the response variable, followed by a simple prediction model (for instance, *y = u + g + e*), may lead to loss of information. The results of this study showed that the predictive ability of ssGBLUP was acceptable and reliable in both the random cross-validation and forward prediction scenarios and was comparable to the predictive ability obtained in a previous study on piglets born alive [8]. Compared with GBLUP, the predictive ability of ssGBLUP was better. 

A simple method was used to blend the pedigree-derived relationship matrix and the marker-derived relationship matrix to construct the **H** matrix (i.e., **G**_w_ = 0.9**G** + 0.1**A**_22_). However, an alternative parameter setting, such as a grid search [27], could be used to obtain better predictive ability. In addition, weighted ssGBLUP [28] should also be considered in future studies to account for the locus-specific or genomic-window-specific variances [29] and thus gain better predictive accuracy using trait-specific prediction models. Previous studies have reported the superiority of weighted ssGBLUP using both simulations [28] and dairy goat data [12,30] with respect to the predictive accuracy. Moreover, under the framework of weighted ssGBLUP, marker weights can be assigned according to preidentified QTL regions [7] or tightly associated markers [31]. In addition, the simple weighting approach used in this study provided preferable or at least comparable predictive ability with regard to the traits that were studied.

## 5. Conclusions

In conclusion, the sperm morphology abnormality traits included in this study have low to moderate heritability. Both GBLUP and ssGBLUP showed comparative predictive abilities, and ssGBLUP outperformed GBLUP under many circumstances. To our knowledge, this is the first time that the genetic parameters and genomic predictive ability of these semen traits were reported in such a large Duroc boar population.

## Figures and Tables

**Table 1:** Descriptive statistics of sperm morphology abnormalities in the Duroc boar population.

Traits ^1^	#obs. ^2^	#indiv ^3^	Min.	Mean	Max.	SD	CV (%)
CT (%)	29,526	1304	0	0.15	5.3	0.24	162.22
BT (%)	29,526	1304	0	2.41	48	2.12	88.17
PD (%)	29,526	1304	0	4.94	41.7	3.07	62.12
DD (%)	29,526	1304	0	7.25	50.4	4.77	65.73
DMR (%)	28,973	1232	0.10	2.82	38.1	2.58	91.22

^1^ CT: Coiled tail; BT: Bent tail; PD: Proximal droplet; DD: Distal droplet; DMR: Distal midpiece reflex. ^2^ The number of phenotypic observations, #. denotes “the number of individuals”. ^3^ The number of boars with phenotypic records.

**Table 2:** Data structure of the training and test population in the forward prediction scenario. Boars from the 1st to 9th and the 10th to 11th generations were treated as training and test groups, respectively.

Dataset	#. Pedigree ^1^	#. Genotypes ^2^	#. Phenotypes ^3^	#. Pheno & Geno ^4^
Training	4256	1247	1012	657
Test	1028	376	292	172

^1^ The number of pigs in the pedigree, #. denotes “the number of individuals”. ^2^ The number of pigs with genotypes. ^3^ The number of boars with phenotypic records. ^4^ The number of boars with both phenotypic records and genotypic data.

**Table 3:** Genetic parameters for five types of sperm morphology abnormalities estimated by a single-trait linear mixed model in the Duroc boar population.

Traits ^1^	σ_a_^2^	σ_p_^2^	σ_e_^2^	h ^2^ (SE)	r (SE)
CT	0.0016	0.0047	0.0494	0.0287 (0.0106)	0.1133 (0.0068)
BT	0.5960	0.6929	3.0432	0.1376 (0.0299)	0.2975 (0.0123)
PD	2.5734	3.2141	4.7412	0.2444 (0.0453)	0.5497 (0.0131)
DD	7.2729	4.2530	13.1157	0.2951 (0.0453)	0.4677 (0.0147)
DMR	2.2786	2.8257	3.4195	0.2673 (0.0481)	0.5988 (0.0127)

^1^ CT: Coiled tail; BT: Bent tail; PD: Proximal droplet; DD: Distal droplet; DMR: Distal midpiece reflex; σ_a_^2^: additive genetic variance; σ_p_^2^: permanent environmental variance; σ_e_^2^: residual variance; h^2^: heritability; r: repeatability; SE: standard error.

**Table 4:** Genetic correlation (upper triangle), covariance (lower triangle), and heritability (diagonal) of sperm morphology abnormalities estimated by a five-trait linear mixed model in the Duroc boar population (estimate (SE)).

Traits ^1^	CT	BT	PD	DD	DMR
CT	0.036 (0.011)	0.955 (0.027)	0.329 (0.158)	0.207 (0.161)	0.403 (0.159)
BT	0.033 (0.009)	0.134 (0.029)	0.325 (0.134)	0.305 (0.131)	0.378 (0.134)
PD	0.023 (0.014)	0.394 (0.199)	0.239 (0.045)	0.607 (0.091)	0.052 (0.142)
DD	0.025 (0.020)	0.631 (0.301)	2.609 (0.639)	0.297 (0.045)	0.207 (0.123)
DMR	0.027 (0.012)	0.434 (0.181)	0.124 (0.345)	0.845 (0.550)	0.264 (0.048)

^1^ CT: Coiled tail; BT: Bent tail; PD: Proximal droplet; DD: Distal droplet; DMR: Distal midpiece reflex.

**Table 5:** Predictive abilities for sperm morphology abnormalities in the Duroc boar population.

Traits ^1^	Cross-Validation		Forward Prediction	
	GBLUP	ssGBLUP	GBLUP	ssGBLUP
CT	0.212 ± 0.004	0.249 ± 0.004	0.069	0.085
BT	0.373 ± 0.003	0.439 ± 0.003	0.311	0.311
PD	0.330 ± 0.003	0.565 ± 0.002	0.250	0.311
DD	0.386 ± 0.003	0.507 ± 0.002	0.389	0.442
DMR	0.417 ± 0.003	0.561 ± 0.002	0.344	0.483
Average	0.344	0.464	0.273	0.326

^1^ CT: Coiled tail; BT: Bent tail; PD: Proximal droplet; DD: Distal droplet; DMR: Distal midpiece reflex; GBLUP: genomic best linear unbiased prediction; ssGBLUP: single-step GBLUP.
